# Peracetic acid effects on human bronchial cells in an air liquid interface

**DOI:** 10.1371/journal.pone.0322926

**Published:** 2025-05-05

**Authors:** Ellie S. Burns, Nicole S. Olgun, Sherri A. Friend, Walter G. McKinney, Erik W. Sinsel, Jeffrey S. Reynolds, Stephen S. Leonard

**Affiliations:** 1 Health Effects Laboratory Division, National Institute for Occupational Safety and Health, Morgantown, West Virginia, United States of America; 2 Gradient, Charlottesville, VA, West Virginia, United States of America; University of Kansas Medical Center, UNITED STATES OF AMERICA

## Abstract

Peracetic acid (PAA) is an organic peroxide commonly used as a disinfectant or sterilizing agent across many industries such as in agriculture, water treatment plants, and healthcare facilities. PAA is versatile, effective, and is considered environmentally friendly due to its decomposition products which include acetic acid, oxygen, and water. However, occupational researchers recognize that it is also highly corrosive as well as a strong oxidizer, and exposure to peracetic acid can severely irritate the respiratory system and this mixture can even act as an asthmagen. To determine the effect of PAA exposure to human airways, normal human bronchial epithelial cells (NHBE) were cultured at the air liquid interface and then exposed to PAA vapors generated across four separate concentrations: 0, 3, 12, and 24 ppm during four-hour exposure periods. Cells were allowed time to recover post-exposure for four and twenty-four hours prior to analysis. Cellular response and toxicity were assessed through metabolic assays for cell viability and cytotoxicity, cell layer integrity (using transepithelial electrical resistance (TEER) measures), and ELISA assays for endothelin-1 (ET-1) (pro-inflammatory mediator and vasoconstrictor) and pro-inflammatory cytokines (IL-6, IL-8). Histological changes were examined for presence of mucosubstances and overall tissue layer structure. PAA exposure had a significant effect on cytotoxicity wherein cytotoxic effect increased with dose concentration and recovery duration. Conversely, cell viability decreased significantly with dose and recovery period. Furthermore, exposure to PAA lowered transepithelial resistance significantly between controls and exposure conditions. ET-1, IL-6, and IL-8 were also assessed from culture fluid and were found to respond to dosage and recovery length. Histology suggested an injury response and cell layer disruptions at 12 ppm exposure and showed indicators of cell death at 24 ppm. Our findings suggest that PAA induces cell damage and a pro-inflammatory response in human bronchial cells with increasing dose and recovery time that reflects increased cell mortality at higher concentrations. Future work will extend this study to the human nasal epithelium to discern health effects across airway tissues.

## Introduction

Peracetic acid (PAA), or peroxyacetic acid, is a highly reactive organic peroxide compound used as a common sterilizer and disinfectant across healthcare, water treatment, and food processing industries among other commercial applications due to its effectiveness and its decomposition byproducts [1–5]. In solution with water, PAA exists in an equilibrium with hydrogen peroxide (HP) and acetic acid (AA). The strong reducing potential of PAA is effective in disrupting organic macromolecules, leading to cellular death without damaging sensitive, critical, or semi-critical equipment such as endoscopes or other surgical devices [6–8].

PAA-HP mixtures have been recognized as an asthmagen by the Association of Occupational and Environmental Clinics (AOEC) [9]. Occupational exposure to PAA has been linked to acute airway inflammation and asthma-like symptoms, including ocular, nasal, throat, and/or skin irritation, wheezing, shortness of breath, and cough [2,6,10–13]. Occupationally associated symptoms and sensitization can be exacerbated by frequent, repeated exposures but may resolve after discontinuation of use [6,11]. Conversely, continued use of an asthma-related occupational hazard when sensitized has been noted to increase the severity of effects [14,15]. Due to the presence of AA and HP within PAA mixtures, both exposures and resultant symptoms are considered in conjunction with these constituents rather than from PAA alone as both HP and AA are recognized as sensory irritants. Investigations of relative sensitivity and response between PAA mixtures and HP or AA individually reveal a much lower threshold of response to PAA than its counterparts, suggesting it may drive more of the irritation responses observed during and after workplace exposure [16].

When sold as an industrial cleaning solution, formulations may contain up to 40% PAA and typically require dilution before use. Both dilution and sterilization have been noted as tasks capable of generating occupational exposures to PAA [2]. The specific cleaning activity or disinfection level (i.e.,; surgical sterilization and sporicidal decontamination versus general, low-level disinfection) can determine the application strength or duration and thus potential exposure intensity [7,17]. There are no current published regulatory occupational exposure limits for PAA solutions, however the American Conference of Governmental Industrial Hygienists (ACGIH) recommends a threshold limit value-short term exposure limit (TLV-STEL) of 1.2 mg/m^3^ (0.4 ppm) [18]. Various organizations have proposed different benchmarks for occupational PAA exposures; STELs have been proposed for PAA anywhere from 1.2–1.56 mg/m^3^ for 15-minute short term exposure windows, while shift-length time-weighted averages (8-hour days) have been proposed across the 0.2–0.62 mg/m^3^ range [19].

To examine acute respiratory effects of PAA exposure in human tissues, *in vitro* methodologies were applied to assess the cellular outcomes after vapor exposure at three different concentrations (3, 12, and 24 ppm) based on a concurrent animal exposure series. Results from published mouse studies have used 24 ppm PAA as an “at use” concentration, generating PAA vapor atmospheres directly from buffered commercial product for the maximum exposure threshold [16]. Similarly, the upper dose here continues the use of 24 ppm PAA as a maximum dose value, with middle and low doses designed to capture a spectrum of potential human response effects. Based upon occupational observations, severity of cellular response was expected to increase along PAA vapor concentrations. Normal Human Bronchial Epithelial (NHBE) cells cultured at the Air-Liquid Interface (ALI) create a pseudostratified epithelial layer containing ciliated and goblet cells that more accurately mimics the physiology of the human respiratory tract lining – including mucous production – and allows for a more realistic deposition model in chambered vapor exposure studies. Cellular response and metabolism were examined at four- and twenty-four-hours post-exposure to assess viability and cytotoxicity as well as epithelial layer integrity and markers for pro-inflammatory and injury response.

## Materials and methods

### Cell culture

Normal Human Bronchial Epithelial (NHBE) B-ALI cells were acquired from Lonza (CC-2540S), cryopreserved at passage 2 and stored in liquid nitrogen. Informed consent and authorization for collection of donor bronchial cells for research applications was conducted by the commercial supplier and as such did not require further internal ethics approval. Cells were thawed and expanded in PneumaCult-Ex Plus complete basal medium (Stemcell Technologies) per manufacturer’s specifications under incubation conditions of 37^o^C and 5% CO_2_ (HERACell Cell Locker Incubator, ThermoFisher Scientific). After 4 days, NHBE cells were detached using the Stemcell Animal-Component Free Cell Dissociation Kit (Stemcell Technologies) and seeded along Co-Star 12-well transwell membranes (Corning) at a density of 1.5 x 10^5^ cells per insert (~1.34 x 10^5^ cells/ cm^2^). Cells were then maintained while submerged in PneumaCult-Ex Plus culture medium until >80% confluency, which routinely occurred approximately 7 days after transwell seeding or 11 days after initial seeding. To begin the airlift phase and induce differentiation, apical medium was removed and the medium within the basal chamber was transitioned to PneumaCult-ALI complete base medium (Stemcell Technologies). ALI medium in the basal chamber was changed three times per week with an apical surface 1x PBS (Mg and Ca free) wash once per week until NHBE cells had differentiated into a mature, mucous-producing, ciliated epithelium, roughly 21–28 days after airlift initiation (S1 Fig).

### Peracetic acid vapor generation and exposure system

The PAA vapor was generated for this system in a manner similar to that described by Doepke, et al. (2021) [20]. Laboratory air was passed through a dryer, a charcoal filter, and finally a high efficiency particulate air (HEPA) filter. This filtered air, at laboratory temperature (~68 F), was then delivered to two mass flow controllers (Alicat Scientific, Inc.) configured in parallel: one for PAA vapor generation and one for dilution air. The first mass flow controller supplied dilution air at a fixed 25 L/min then combined with the second mass flow controller that supplied 0–5 L/min of air over the headspace of a room-temperature polytetrafluoroethylene (PTFE) vial containing 5 mL of commercially available PAA solution (32% peracetic acid by weight in dilute acetic acid, Sigma Aldrich product #269336). Initially, the PAA solution was loaded into a syringe which was placed in a computer-controlled syringe pump. At the start of an exposure, the solution was injected into the vial. Following exposure, the solution was pulled back into the syringe. During the exposure, the 0–5 L/min mass flow controllers setpoint was updated every 30 seconds by custom software written in LabView 2021 (National Instruments; Austin, TX). The flow setpoint was determined by a proportional-integral-derivative (PID) feedback control algorithm based on real time readings from a PAA vapor monitor (Interscan, 4000 Series Digital and GasD 8000 Series Portable Gas Analyzers). After mixing of the two mass flow controllers, the PAA vapor was then passed into the top of the exposure chamber through PTFE tubing which was used for all air flow containing PAA.

The exposure chamber used in this system was the Cube 150, which was previously described and tested by Goldsmith et al. (2011) [21]. Briefly, the Cube 150 is an airtight 22 x 22 x 20 inch (L x W x H) exposure chamber constructed from 16 gauge stainless steel with a clear polycarbonate door. A rack for holding animals or cell cultures rested on support beams which were 3/8” outside diameter stainless steel tubes with small holes (0.13-inch diameter) drilled into the undersides that acted as the exhaust pathway for the chamber. Air entered from the top center of the chamber through a dispersion nozzle and was exhausted through the tubes and a carbon/HEPA filter bank to the laboratory exhaust. The Cube 150 was previously tested for concentration homogeneity within the animal cage partitions using aerosol and was reported to have less than 5% variation between all locations within the chamber (data not shown). Inside surfaces of the chamber were lined with thin PTFE sheeting to reduce PAA vapor reaction. Schematic of the exposure chamber set up can be found in S2 Fig.

Real-time PAA concentration was monitored inside the chamber with an Interscan PAA monitor (Interscan Corporation) which was calibrated using a single-analyte photometer (Chemetrics I-2020 Peracetic Acid). PAA concentration was controlled by adjusting the air flow over the PAA vial head space. The temperature and relative humidity inside the exposure chamber were measured and recorded continuously. To maintain the target relative humidity, a solenoid valve connected to a water reservoir was activated by the custom control software to add water to a heated absorbent pad at the base of the chamber to raise and maintain the relative humidity at 90%.

### Peracetic acid vapor exposures

Cells were exposed to 3, 12, and 24 ppm PAA vapors in addition to negative control cells which were exposed to filtered air. PAA vapor was generated and monitored over 4 hours, with a 3.5-hour active exposure period immediately followed by a 30-minute chamber exhaust window before cell retrieval. Cells were transferred to fresh, prewarmed culture medium and then into a cell culture incubator (37^o^C, 5% CO_2_) to recover for either 4 or 24 hours prior to analysis. Each exposure and recovery condition were assessed across three biological replicates (n = 3) consisting of NHBE B-ALI cells cultured across independent plates; separate plates were allocated for four- or twenty-four-hour recoveries, respectively, so that data between time points originated from the same experimental exposure event but 24-hr samples did not experience fluctuations in environmental conditions during 4-hr plate processing. Colonized transwell membrane inserts were randomly allocated for histology, cellular viability, and transepithelial electrical resistance assays. Basolateral culture supernatant was drawn for lactate dehydrogenase and sandwich ELISA assays. Intra-assay variability was assessed by dividing biological samples into a minimum of three technical replicates.

### Cellular viability (MTT assay)

Cellular viability assays were conducted using 0.5 mg/mL MTT (3-(4,5-dimethylthiazol-2-yl)-2,5-diphenyltetrazolium bromide) (Invitrogen) at a volume of 1 mL/cm^2^ [22]. The production and subsequent solubilization of formazan crystals utilizes the redox capacity of viable cells to produce a colorimetric shift that is then quantifiable by microplate reader. In addition to PAA vapor exposures, NHBE cells were exposed to hexavalent chromium (Cr(VI)) as a positive control material. Cell culture transwells were incubated with MTT solution for 4 hours to allow conversion of soluble MTT to formazan before the addition of 1 mL DMSO to solubilize formazan crystals. For each biological sample, 100 µ L of supernatant was transferred to a 96-well plate in triplicate and absorbance read at 540 nm on an H1 Synergy Microplate Reader (Bio-Tek). Percent viability was calculated from the MTT activity derived from the respective filtered air controls for each exposure series.

### Percent cytotoxicity

Cell death was inferred by quantifying lactate dehydrogenase (LDH) activity in basolateral culture supernatant using the Roche LDH Assay Kit (Sigma-Aldrich). LDH reduction of NAD+ results in the production of formazan salts which produce a colorimetric shift that allows for an absorbance-mediated calculation of LDH activity in solution. In conjunction with PAA exposures, as conducted above for viability, Cr(VI) was employed as a positive control to illustrate impacts of a known cytotoxic material in ALI culture. High controls were generated by incubating cell cultures with Triton-X 1000 to induce complete (100%) cytotoxicity. Low controls consisted of cell cultures incubated with culture medium alone to quantify background LDH release in control cell populations. Absorbance readings were taken at 492 nm on an H1 Synergy Microplate Reader (Bio-Tek) and used to calculate percent cytotoxicity of samples (Cytotoxicity Detection Kit, Roche). Assay medium alone was measured and used in background correction of all absorbance readings. Percent cytotoxicity was calculated by subtracting low control absorbance from experimental values and dividing that number by the value obtained from low control correction of the high controls, then multiplied by 100 to obtain percentage.

### Transepithelial electrical resistance

Transepithelial electrical resistance (TEER) measurements were taken in triplicate per sample using an Evom2 Epithelial Voltohmmeter with an STX2 chopstick probe (World Precision Instruments, Inc.) at room temperature. The probe electrodes were placed in the apical and basal chambers, respectively, to assess the resistance in Ohms across the epithelial layer. Reduced epithelial layer thickness or damage to the monolayer reduces the resistance provided by the colonized transwell surface. Blank values were obtained in a well containing an uncolonized transwell membrane.

### Histology

For histological investigations, cells were retrieved after the 24-hour recovery period for fixation. Per replicate, a transwell insert was rinsed with sterile 1x PBS (Ca and Mg free) before fixation in 4% paraformaldehyde for 15 minutes (conducted according to Corning). Inserts were rinsed in 1x PBS once more before storage in 70% ethanol at 4^o^C prior to processing. Cell layers were embedded in paraffin and sectioned before staining with hematoxylin and eosin (H&E) or periodic acid-Schiff (PAS) stain, which were used to detect cellular structure (nuclei, cytoplasm, and extracellular matrices) and mucosubstances, respectively.

### ELISA assays

Sandwich ELISA assays were used to assess extracellular concentrations of Endothelin-1 (ET-1) (Abcam, Endothelin-1 ELISA Kit), Interleukin-6 (IL-6) (Meso Scale Discovery, V-Plex ELISA) and Interleukin-8 (IL-8) (Meso Scale Discovery, V-Plex ELISA). These targets were chosen to represent distinct mechanisms of airway injury response such as potential airway restriction (ET-1), acute phase proinflammatory response (IL-6), and neutrophil recruitment (IL-8). Cell culture supernatant was incubated with primary antibodies before secondary antibody and detector incubations. ET-1 ELISA results were read immediately at 450 nm on a H1 Synergy microplate reader (Bio-Tek) with correction at 580 nm. V-plex ELISA kits for IL-6 and IL-8 were read immediately on a MESO plate imager (Meso Scale Discovery).

### Statistical analysis

Statistical analyses were conducted in R through RStudio (ver. 2024.04.1 Posit Software, PBC). Data was first assessed for normality using Shapiro’s test and Q-Q Plots. Statistical significance (p < 0.05) was determined between test groups and controls as well as between recovery periods within test groups using two-way ANOVA and Tukey’s HSD analyses when appropriate.

## Results

### Peracetic acid exposures reduce cellular viability

**Fig 1:**
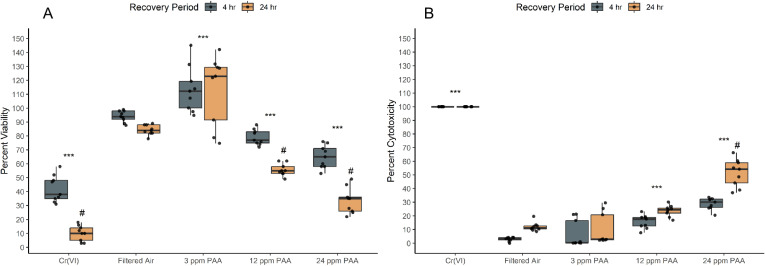
Percent viability and cytotoxicity of NHBE cells are significantly altered by PAA exposure. (A) Viability was measured through formation of formazan crystals during the MTT assay as a metric of living cell metabolism. Percent viability significantly decreased with PAA concentration and between recovery times compared to controls. (B) Cytotoxicity was assessed through production of lactase dehydrogenase (LDH); percent response significantly increased with concentration. Hexavalent chromium (Cr(VI)) was employed as a positive control exposure to compare the impacts of varying PAA vapor concentrations to a known, highly cytotoxic substance. A dose and recovery-based response was observed in both assays in bronchial cell response to PAA. A significant difference within treatments and between recovery times is represented by # (p < 0.05) while a significant difference between a treatment condition and filtered air control is represented by asterisks (* = p < 0.05; ** = p < 0.005; *** = p < 0.0005). Dots overlaid each box plot represent the individually measured data points. Box plot whiskers represent the range of data represented by 1.5x the interquartile range.

Viability of NHBE cell cultures decreased as concentrations of PAA in vapor exposures increased regardless of recovery period; all tested conditions were significantly different compared to filtered air controls (p < 0.05; Fig 1A). Percent viability was significantly lower after 24 hours recovery when compared to a 4-hour sample recovery at both 12 and 24 ppm PAA exposures. At 3 ppm exposure concentrations, culture viability was not significantly different than negative controls. Some percent values were observed above 100%; this is likely due to differences in temperature and humidification between filtered air and PAA vapor generation chamber operations. During initial optimization, it was noted that the four-hour exposure period was the limit before NHBE ALI cultures began to exhibit decline under control, filtered air conditions.

### Peracetic acid vapor induces cytotoxicity at elevated concentrations

Complementary to viability results, a significant increase in percent cytotoxicity occurred at PAA vapor concentrations of 12 and 24 ppm (p < 0.05; Fig 1B) as evidenced by the decline in percent LDH activity. An increased post-exposure duration (24 hours) only significantly increased cytotoxicity at the highest tested PAA exposure concentration (24 ppm; Fig 1B).

### Transepithelial Resistance (TEER)

**Fig 2:**
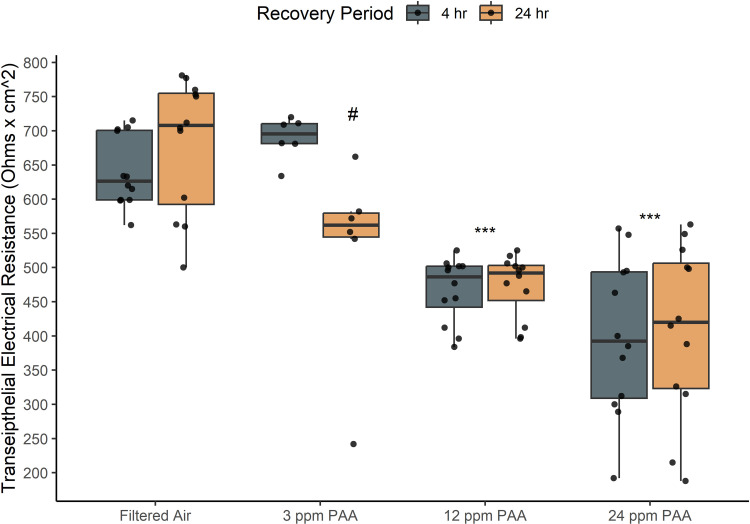
Transepithelial resistance of NHBE decreases significantly after PAA exposure. Transepithelial resistance (TEER) correlates with cell layer integrity; TEER measurements of NHBE cells were significantly reduced at 12 and 24 ppm PAA exposures when compared to the control (***; p < 0.0005). At 3 ppm PAA, TEER was significantly reduced only after 24-hr recovery (#). Dots overlaid each box plot represent the individually measured data points. Box plot whiskers represent the range of data represented by 1.5x the interquartile range.

TEER measurements indicated significantly reduced epithelial resistance between 12 and 24 ppm PAA exposures and the negative control (p < 0.05; Fig 2) but epithelial resistance at 3 ppm PAA was not significantly different from filtered air overall. Additionally, recovery duration did not significantly impact epithelial electrical resistance between PAA treatments at high doses apart from significantly lowered resistance observed in 3 ppm PAA treatments after 24-hours.

### Histology

**Fig 3:**
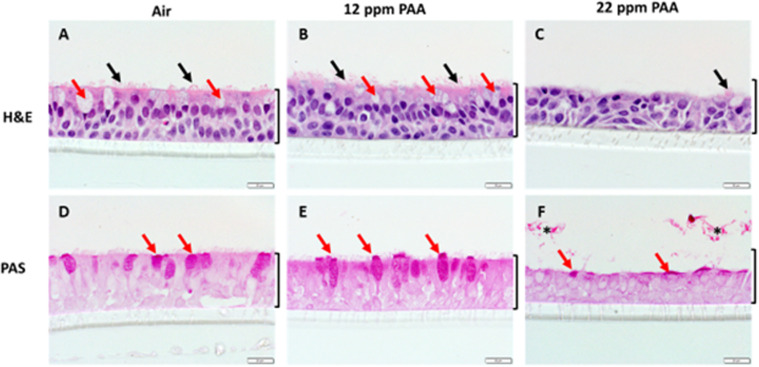
Histological staining and cell microscopy reveals cellular disruption and death after application of PAA and 24-hr recovery. Hematoxylin and Eosin (A-C) staining and PAS staining (D-F) reveal cilia (black arrows) and goblet cells (red arrows). 12 ppm PAA-exposed cell layers (B, E) indicate injury response in NHBE through a loss of cilia (B) and increased goblet cell production (E). After 24 ppm PPA exposure, staining shows ablation of the apical cell layer, irregular nuclei, and epithelial cell blunting (C,F).

A qualitative assessment of cell layer structure and integrity was conducted through H&E and PAS staining of ALI cross-sections after exposures of filtered air, 12 ppm, and 24 ppm PAA which were fixed after 24 hours of recovery time (Fig 3). Observations of culture morphology were consistent across all replicates. Filtered Air controls produced a robust, ciliated epithelium with goblet cells and round nuclei throughout the epithelial layer (Fig 3A and 3D). At 12 ppm, damage to the cilia is observable in H&E-stained sections (Fig 3B), as well as minor nuclear disorganization. In PAS-stained sections (Fig 3E), surface level mucosal secretion can be observed as well as a proliferation in the apical layer of goblet cells. After 24-ppm PAA exposures, ablation of the apical layer is prominent; both goblet cells and cilia are no longer visible (Fig 3C and 3F). In H&E-stained sections (Fig 3C), nuclei appear irregular and disorganized.

### Endothelin-1 (ET-1)

**Fig 4:**
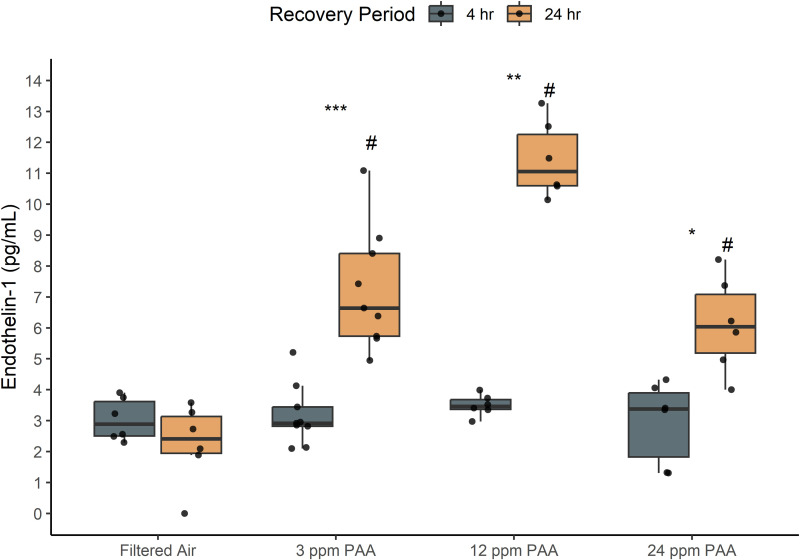
Endothelin-1 production significantly increases in 24-hour recoveries post-PAA exposure. All exposure concentrations of PAA induced a significant increase in ET-1 concentration after 24-hour recovery compared to filtered air. A significant difference within treatments and between recovery times is represented by # (p < 0.05) while a significant difference between a treatment condition and filtered air control is represented by asterisks (* = p < 0.05; ** = p < 0.005; *** = p < 0.0005). Dots overlaid each box plot represent the individually measured data points. Box plot whiskers represent the range of data represented by 1.5x the interquartile range.

ET-1 production increased significantly between filtered air and each PAA exposure concentration (p < 0.05; Fig 4). In each case, this significant difference occurred due to elevation of ET-1 only after 24-hour recoveries; the protein concentrations for each treatment did not differ significantly between groups at the 4-hour post-exposure assessment.

### Interleukin-6 and Interleukin-8 vary in response trends

**Fig 5:**
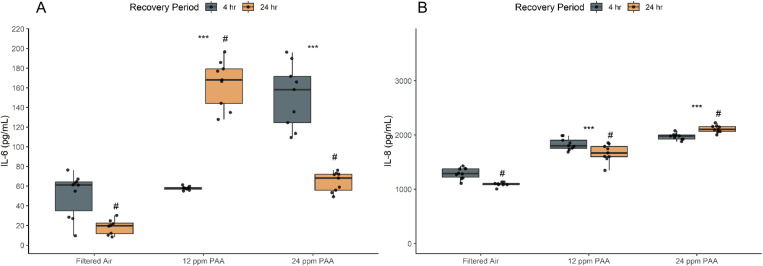
Cytokine response is elevated in NHBE cells after PAA exposure. IL-6 (A) and IL-8 (B) increase significantly after PAA exposure, suggesting elevated inflammatory response and immune cell recruitment at 12 and 24 ppm PAA. After exposure to 24 ppm PAA, IL-6 concentration (A) is comparatively reduced at 24-hrs post-exposure suggesting response fluctuates with cell viability. A significant difference within treatments and between recovery times is represented by # (p < 0.05) while a significant difference between a treatment condition and filtered air control is represented by asterisks. (* = p < 0.05; ** = p < 0.005; *** = p < 0.0005). Dots overlaid each box plot represent the individually measured data points. Box plot whiskers represent the range of data represented by 1.5x the interquartile range.

IL-6 and IL-8 concentration increased significantly at 12 and 24 ppm PAA treatments when compared to filtered air controls (p < 0.05; Fig 5A and 5B). In both PAA treatment conditions, IL-6 concentration differed significantly between recovery periods (Fig 5A); recovery increased IL-6 concentration between 4- and 24-hour recovery at 12 ppm PAA while recovery decreased protein concentration along recovery duration after 24 ppm PAA exposures. Conversely, in assessments of IL-8 response, protein concentration significantly increased along recovery duration for 24 ppm PAA exposures (Fig 5B).

## Discussion

In vitro data reported in this study suggest that adverse cellular outcomes to PAA vapor exposures increase with exposure concentration as well as post-exposure recovery duration. Occupational reports and case studies regarding workplace exposures to PAA-HP mixtures report airway, nasal, skin, and/or ocular irritation, cough, wheezing, shortness of breath, and general asthma-like symptoms [2,6,10–13]. In most reported case studies of PAA-induced sensitization and occupational asthma, these symptoms often abate with time after exposures to the PAA cleaning mixture cease [6,11]. As noted previously, the lack of harmful degradation products often positions peracetic acid mixtures as a favorable alternative to other disinfectants, but this does not denote a lack of risk to human health. Murine sensory irritation data generated by Gagnaire et al. (2002) described a 50% reduction in respiratory rate (RD_50_) at 5.4 ppm PAA which was likened to the irritant potential of chemicals such as formaldehyde and chlorine [16]. In comparison, within this same study, the RD_50_ of HP and AA were estimated to be 123 and 227 ppm, respectively, which suggests a much lower comparative irritancy risk, though not insignificant [16].

Occupational exposures for PAA vary with application strength, duration, and frequency of disinfectant tasks. The data generated here represents a single exposure to a naïve cell population; exposures within occupational settings are anticipated to be repetitive which, in combination with other common sterilizing agents, may create a sensitization effect over time. While the upper range of these *in vitro* dosages may be greater than that measured in some occupational environments, these results may instead be better viewed through the lens of either high-dose exposures or an estimation of potential cumulative respiratory effects. Additionally, implementation of a physiologically relevant *in vitro* model allows us to test elevated concentrations in bronchial epithelia without undue risk, thereby highlighting potential of worst-case outcomes. Limitations common to *in vitro* models such as these often include reduced sample size and biological diversity when compared to occupational or other human exposure studies. With this in mind, this study aims not to explore the extent of human bronchial sensitivity to PAA vapor but to describe general trends in cellular response along a spectrum of PAA concentrations and common human biomarkers relevant to culture integrity, inflammation, and immune response. These data indices of airway irritation were conducted to supplement ongoing health hazard characterization of PAA vapor exposures within our institute. For the consideration of exposure guidance, these *in vitro* investigations should be expanded both in sample size and donor pool to more accurately describe the potential spectra of human dose response.

These data corroborate the expected link between concentration and negative cellular impacts. Cellular viability decreased in bronchial epithelia with PAA concentration and over recovery time which suggest deleterious effects after PAA mixture exposure continue to negatively impact cellular metabolism from the four-hour to twenty-four-hour post-treatment time points. While LDH release – and thus inferred cytotoxicity – significantly increased with dose, post-exposure recovery length effects were only observed at the highest dose (24 ppm). These combined results indicate that PAA impacts cellular metabolism and viability in a dose and time-dependent manner that suggests effects scale with dose and may delay onset or accelerate symptom development in the hours post-exposure.

A decrease in TEER measurement indicates reduced cell layer integrity and increased permeability following exposure but these data did not discern further differences between higher doses. Histological analysis corroborated the reduction in epithelial integrity observed in TEER analysis through visible injury response and cell layer disruptions at 12 ppm exposure and, further, cell death and apical layer ablation at 24 ppm as indicated by cell debris and reduced cell layer thickness of cross-sections. This may suggest that although exposures to mixtures from 12 to 24 ppm PAA do illustrate a relatively consistent reduction in resistance measurements beyond a certain level of epithelial damage, qualitatively observed physiological impacts may be more severe as exposure dose increases. Notably, TEER measurements did not decrease significantly between recovery times except at 3 ppm exposures, although recovery period did not significantly impact cytotoxicity and viability data at this dosage. Only some endpoints measured at 3 ppm were significantly different from the negative control which suggests that this dose range begins to demonstrate initial disruption of cell metabolism and epithelial health and, most often, these effects are most evident after a delayed period post-exposure (24-hours).

ET-1 production in bronchial epithelium and endothelium is attributed to increased vasoconstriction or bronchoconstrictive response when bound along distinct respiratory receptors across adjacent airway tissues (ET_A_ and ET_B_); action of ET-1 is associated with COPD, asthma, and pulmonary hypertension [23–25]. While this study did not investigate receptor binding and therefore is ambiguous as to specific protein activity, varied ET-1 response to PAA corresponds to a dose-dependent potential for further downstream effects. A reduced concentration of ET-1 after 24 ppm PAA exposures is likely a function of fewer living cells; increased cytotoxicity and reduced viability indicate a lower proportion of living cells at higher exposure concentrations which may reduce the potential to generate a proportional ET-1 response. ET-1 levels increased significantly between 4- and 24-hour recovery cohorts despite increased cytotoxicity and reduced viability within the 24-hour recovery populations. Together, these data suggest that potential bronchoconstriction is delayed but exposure produces a consistent, significant increase in protein levels after all tested exposure concentrations.

Lastly, trends in interleukin response varied between IL-6 and IL-8, likely as these mediators operate in separate pathways of respiratory injury response. In respiratory epithelia, IL-6 is associated with acute phase inflammatory responses, pro- and anti-inflammatory signaling, as well as lymphocyte and macrophage recruitment [26,27]. Conversely, IL-8 is associated with immune and inflammation response through neutrophil recruitment and activation in the respiratory lining [28–30]. The varied protein concentrations observed in IL-6 and IL-8 across dose and recovery lengths is likely to stem from functional differences in human immunity and inflammation. IL-8 response increased significantly with dose but showed sustained and increased protein concentrations even at 24-hours post-exposure to 24 ppm PAA. However, at the same dose and recovery duration, IL-6 concentrations fell significantly when compared to protein levels 4-hours post-exposure which appeared to correspond with the cytotoxicity and viability trends at these periods.

## Conclusions

Overall, our findings suggest that PAA induces cell damage and an inflammatory response in human bronchial cells in a dose and time dependent manner. These data further suggest that some effects – such as changes in viability, cytotoxicity, and protein concentrations – are more pronounced 24-hours after exposure which implies that airway impacts may begin at exposure but may not reach full severity until after the fact.

*The findings and conclusions in this report are those of the authors and do not necessarily represent the official position of the National Institute for Occupational Safety and Health (NIOSH), Centers for Disease Control and Prevention (CDC). Mention of any company or product does not constitute endorsement by NIOSH, CDC.*

## Supporting Information

S1 Fig Structure of mature Normal Human Bronchial Epithelium cultured at the Air Liquid Interface. Electron micrograph of mature NHBE B-ALI epithelial cross-section depicts A) ciliated epithelium (red arrow), B) goblet cells (blue arrow), nuclei (green arrow), and tight junctions (yellow arrow).
(TIF)

S2 Fig Diagram of PAA vapor generation and exposure scheme. Mass flow controllers (labeled MFC) regulated mixing of filtered air which was directed over the headspace of a commercial PAA solution vial to generate PAA vapors. Real-time chamber vapor concentration was monitored internally by an Interscan PAA monitor and controlled by air flow adjustments. Temperature and relative humidity were also recorded and monitored continuously. Cell culture plates (representation in blue) were placed on an elevated on a support platform during exposure.
(TIF)
