# Supplementary figures and images for: Peracetic acid effects on human bronchial cells in an air liquid interface
Source: PLoS One. 2025 May 5;20(5):e0322926. doi: 10.1371/journal.pone.0322926 (PMC12052169; doi:10.1371/journal.pone.0322926)

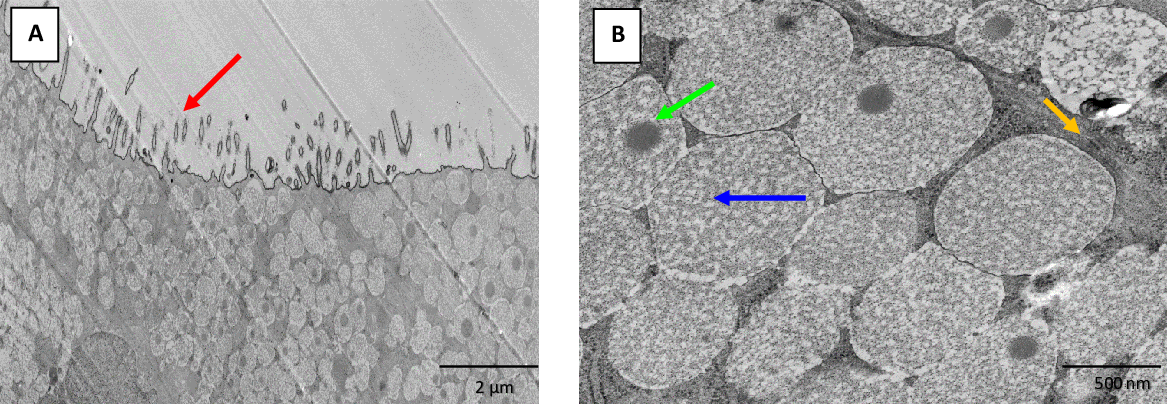

Supplement: S1 Fig — Electron micrograph of mature NHBE B-ALI epithelial cross-section depicts A) ciliated epithelium (red arrow), B) goblet cells (blue arrow), nuclei (green arrow), and tight junctions (yellow arrow). (TIF) [file pone.0322926.s001.tif]

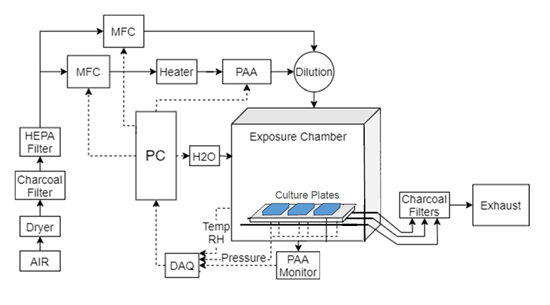

Supplement: S2 Fig — Mass flow controllers (labeled MFC) regulated mixing of filtered air which was directed over the headspace of a commercial PAA solution vial to generate PAA vapors. Real-time chamber vapor concentration was monitored internally by an Interscan PAA monitor and controlled by air flow adjustments. Temperature and relative humidity were also recorded and monitored continuously. Cell culture plates (representation in blue) were placed on an elevated on a support platform during exposure. (TIF) [file pone.0322926.s002.tif]
